# Effect of Oral Ketone Body Intake on Human CD8^+^ T-Cell Immunometabolism

**DOI:** 10.3390/nu18050778

**Published:** 2026-02-27

**Authors:** David Effinger, Simon Hirschberger, Thore Arntjen, Michaela Zell, Lesca Miriam Holdt, Simone Kreth

**Affiliations:** 1 Research Unit Immune Function and Immune Metabolism, Walter Brendel Centre of Experimental Medicine, Ludwig-Maximilian-University (LMU), 81377 Munich, Germany; david.effinger@med.uni-muenchen.de (D.E.);; 2 Department of Anaesthesiology, LMU University Hospital, 81377 Munich, Germany; 3 Institute of Laboratory Medicine, LMU University Hospital, 81377 Munich, Germany

**Keywords:** exogenous ketones, T-cell function, immunometabolism, cytotoxic T cells, ketogenic diet, ketone salts, ketone esters

## Abstract

**Background/Objectives**: The ketogenic diet (KD) has been shown to exert beneficial effects on human immunity by enhancing cytotoxic T lymphocyte function through metabolic reprogramming. However, strict dietary restrictions limit adherence and complicate its use in clinical practice. Exogenous ketone supplements have therefore been promoted as a more feasible alternative to elevate ketone body levels without the need for dietary changes. The objective of this study was to assess whether ketone salt or ketone ester supplementation can reproduce KD-mediated immunometabolic effects on CD8^+^ T cells in healthy individuals. **Methods**: In a prospective interventional study, healthy volunteers received either ketone salts (KS) or ketone esters (KE) for three weeks. Plasma β-hydroxybutyrate (BHB) concentrations were determined, and CD8^+^ T-cell cytokine secretion, functional responses, and mitochondrial energy metabolism were analyzed. In a subgroup, KS supplementation was combined with a carbohydrate-restricted, non-ketogenic diet. **Results**: While KS supplementation resulted in a short-lived increase in plasma BHB concentrations followed by increased BHB uptake in immune cells, KE supplementation led to more sustained plasma BHB levels, however, without detectable intracellular BHB accumulation. Neither intervention affected CD8^+^ T-cell cytokine production, functional capacity, or mitochondrial energy metabolism, and KS intake combined with a carbohydrate-restricted, non-ketogenic diet likewise did not alter CD8^+^ T-cell immunometabolic parameters. **Conclusions**: Transient elevation of circulating ketone body levels through supplementation seems insufficient to reproduce the immunometabolic effects of a KD, which likely require broader metabolic adaptations. Thus, the impact of exogenous ketones on adaptive immunity in healthy individuals appears limited.

## 1. Introduction

The immunomodulatory dietary strategies have gained increasing attention in clinical medicine, as accumulating evidence indicates that environmental nutrient availability profoundly influences immune metabolism and, consequently, T-cell function [1,2,3]. Among these strategies, the ketogenic diet (KD) has emerged as a particular focus of research [4,5,6]. During a KD, endogenous production of ketone bodies, primarily β-hydroxybutyrate (BHB), generated through fatty acid oxidation, not only sustains an adequate energy supply but also modulates immune function by promoting a metabolic shift toward oxidative phosphorylation [7,8]. Our research group has previously demonstrated that a KD significantly enhances CD8^+^ cytotoxic T lymphocyte (CTL) immunity, both in healthy individuals and in patients with COVID-19, and may have the potential to improve clinical outcomes in patients with sepsis [8,9,10].

Although isocaloric very-low-carbohydrate ketogenic diets have been shown to be safe and well tolerated, adherence to a strict ketogenic regimen remains challenging in both clinical and everyday contexts due to its considerable dietary restrictions [11,12]. Successful implementation requires not only structured nutritional counseling but also sustained patient compliance to maintain carbohydrate intake below the ketogenic threshold. To overcome these limitations, exogenous ketone bodies have emerged as a promising nutritional intervention, frequently promoted as a convenient and rapidly effective adjunct—or even an alternative—to the ketogenic diet. These BHB-based compounds are designed to elevate systemic ketone levels without necessitating major dietary modifications. Upon oral administration, exogenous BHB enters metabolic pathways analogous to those of its endogenous counterpart, contributing to cellular energy production via the citric acid cycle. Preliminary evidence further suggests that exogenous BHB may exert stabilizing effects on glucose, insulin, and triglyceride homeostasis [13,14,15].

However, it remains unclear whether oral supplementation with exogenous ketones elicits immunological effects comparable to those induced by a ketogenic diet. In particular, it is not yet known whether exogenous ketones could serve as a viable clinical tool to modulate human adaptive immunity. To address this knowledge gap, we investigated the impact of exogenous ketones on human CD8^+^ CTL immunometabolism. We conducted a prospective, pre–post interventional study in healthy volunteers, administering either ketone salts or ketone esters over a 3-week period, and assessed the effects of both formulations on immune metabolism and function.

## 2. Materials and Methods

### 2.1. Study Design

A prospective, monocentric, clinical-experimental study was conducted on a cohort of adult healthy volunteers with planned oral intake of exogenous ketone bodies provided as ketone salts KETO//OS NAT, PRUVIT (Ventures Inc., Melissa, TX, USA) or ketone esters △G Ketone Performance (TdeltaS Global, Inc., Orlando, FL, USA) over a period of 3 weeks. According to the manufacturer’s instructions, participants consumed one serving of the respective ketone supplement per intake. A total of two servings per day were administered (one in the morning and one in the afternoon). Blood samples were collected before the first intake (T0) and after three weeks of exogenous ketone salt or ester supplementation (T1). An additional cohort of healthy adults received exogenous ketone salts combined with a carbohydrate-restricted, non-ketogenic diet with blood sampling at the same time points. These study participants received detailed nutritional counseling from a board-certified nutritionist prior to the study and were closely monitored throughout the entire study period. To assess nutrient intake, 24 h diet recalls were conducted in all cohorts, and the macronutrient composition is presented in Supplemental Table S1. Baseline characteristics of participants are shown in Table 1.

The primary study endpoint was the change in peak β-hydroxybutyrate concentrations in capillary whole blood after 3 weeks of ketone body supplementation, measured by point-of-care testing. A priori power analysis was conducted based on previous findings regarding changes in peak capillary BHB concentrations [11]. For sample size estimation, a conservative scenario was assumed, incorporating a 20% reduction in the expected effect (Δ = 2.0 mmol/L) and a 20% increase in variability (SD of paired differences = 1.32 mmol/L). Under these assumptions, the standardized effect size (Cohen’s dz) was 1.52. For a two-sided paired t-test with α = 0.05 and 95% power, 8 evaluable participants were required. To account for an anticipated 20% dropout rate, the total sample size was increased to 10 participants.

Secondary endpoints included analyses of T-cell activation, functional, and metabolic parameters, as well as assessments of body composition and quality of life. Beyond that, analyses not prespecified were considered exploratory. All data were collected at our laboratory facilities at the LMU University Hospital, Munich, between October 2023 and August 2025. The inclusion criteria for the study population were as follows: healthy volunteers aged ≥18 years were eligible for participation. Exclusion criteria included: pregnancy or breastfeeding, severe metabolic disorders (e.g., insulin-dependent diabetes), serious autoimmune diseases, acute infections, and personal relationships with the investigator. Written informed consent was obtained from all volunteers enrolled in the study. Research was performed according to the Declaration of Helsinki (ethical principles for medical research involving human subjects). The study designs and the study protocols were approved by the Institutional Ethics Committee of the Ludwig-Maximilian-University Munich, Germany (No. 23-0372) and the study is registered at the DKRS (German Clinical Trials Register; DRKS00034793). All products were independently financed through university or departmental funds; therefore, no competing interests exist.

### 2.2. Bioelectrical Impedance Analysis

Bioimpedance analysis was conducted using a Tanita MC-780 (Tanita Europe, Stuttgart, Germany) with a multifrequency approach (5 kHz/50 kHz/250 kHz), following the manufacturer’s instructions. Participants were weighed barefoot in light clothing, and during measurements, they maintained an orthostatic position. Prior to the bioelectrical impedance analysis, participants were required to meet the following conditions: (1) fasting state, (2) no intense physical activity in the last 24 h, (3) no alcohol consumption in the last 24 h, and (4) urination immediately before the test. Measurements were taken between 8 a.m. and 10 a.m. BMI was calculated using height, measured with a Soehnle Professional 5003 (Soehnle Industrial Solutions GmbH, Backnang, Germany), according to the manufacturer’s guidelines.

### 2.3. Blood Sampling and CD8^+^ T-Cell Isolation

All blood samples were collected after an overnight fast. Blood was drawn into S-Monovettes (Sarstedt AG & Co. KG, Nümbrecht, Germany) containing lithium heparin for subsequent isolation of peripheral blood mononuclear cells (PBMC) via density gradient centrifugation using Histopaque 1077 (Sigma-Aldrich, St. Louis, MO, USA). Cell count and viability were assessed using a ViCell analyzer (Beckman Coulter, Fullerton, CA, USA). PBMC were cultured in RPMI 1640 (Invitrogen, Carlsbad, CA, USA) supplemented with 10% heat-inactivated fetal calf serum (Biochrom, Berlin, Germany), 1% L-glutamine (Life Technologies, Carlsbad, CA, USA), and 1% HEPES (Sigma-Aldrich, St. Louis, MO, USA) under T cell stimulation using CD3/CD28 Dynabeads (Thermo Fisher Scientific, Waltham, MA, USA) at a bead-to-cell ratio of 1:8. CD8^+^ T cells were isolated from PBMC via microbead-based magnetic separation using the AutoMACS Pro Separator, according to the manufacturer’s protocol (Human CD8 MicroBeads, 130-045-201, Miltenyi Biotec, Bergisch Gladbach, Germany).

### 2.4. Flow Cytometry

Flow cytometric antibody staining was carried out according to the manufacturer’s instructions. CD8^+^ T cells were incubated with the respective antibody for 30 min on ice in the dark (CD25 Antibody, anti-human, Vio^®^ Bright B515, 130-113-849; CD38 Antibody, anti-human, APC, 130-113-991; CD44 Antibody, anti-human, PE, 130-113-904; CD69 Antibody, anti-human, FITC, 130-112-801; all Miltenyi Biotec, Bergisch-Gladbach, Germany). Flow cytometric acquisition was performed using a BD FACSCanto II (BD Biosciences, San Jose, CA, USA), and data were analyzed with FlowJo v10 (FlowJo, Ashland, OR, USA).

### 2.5. RNA Extraction, cDNA Synthesis and Quantitative RT-PCR

Quantification of mRNA expression was performed on a LightCycler 480 platform (Roche Diagnostics, Mannheim, Germany) according to established protocols [16]. RNA was extracted using the miRNeasy Kit (Qiagen, Hilden, Germany) and subjected to DNase treatment on columns. Reverse transcription of equal amounts of RNA was performed using Superscript III (Invitrogen, Carlsbad, CA, USA) together with random hexamers and oligo(dT) primers. TATA box-binding protein (TBP) and Ribosomal Protein L13a (RPL13A) were used as reference genes for normalization. Information on primer assays is listed in Supplemental Table S2. Cq values were determined using the second derivative of the maximum approach implemented in the LightCycler software V 1.5.1.62. Cq values > 40 have been considered unspecific. Duplicate values were used for all analysis.

### 2.6. Enzyme-Linked Immunosorbent Assay (ELISA)

Quantification of secreted proteins was performed using enzyme-linked immunosorbent assays (ELISA) according to the manufacturer’s instructions (IFNγ: #430104; Granzyme B: #439207; Biolegend, San Diego, CA, USA|Perforin: #3465-1HP-2, Mabtec, Stockholm, Sweden). Absorbance was measured using a FilterMax F3 MultiMode Microplate Reader (Molecular Devices, LLC, San Jose, CA, USA), and concentrations were determined using the kit-specific standard curves. All samples were run in duplicates, and mean values were used for analysis.

### 2.7. Cytotoxicity Assay

CD8^+^ T cell-mediated cytotoxicity was assessed using a calcein-acetoxymethyl (AM)–based lysis assay. Target K562 lymphoblasts were labeled with 8 µM calcein AM (Sigma-Aldrich, Darmstadt, Germany) and co-cultured with isolated CD8^+^ T cells. Upon lysis of target cells, calcein was released into the supernatant, and fluorescence intensity was measured using a FilterMax F3 MultiMode Microplate Reader (excitation: 480 nm; emission: 520 nm; Molecular Devices LLC, San Jose, CA, USA). 1% Triton X-100 was used as a positive control (maximum release). Specific lysis was calculated using the following formula: [(test release − spontaneous release)/(maximum release − spontaneous release)] × 100.

### 2.8. Oxygen Consumption Rate (OCR) and Extracellular Acidification Rate (ECAR)

Mitochondrial function and glycolytic activity were analyzed in real-time using the Seahorse HS Mini Analyzer, following the manufacturer’s established protocols (Agilent Technologies, Inc., Santa Clara, CA, USA). CD8^+^ T cells were seeded in poly-L-lysine-coated (Biochrom, #L7240, Berlin, Germany) XFp microplates (Agilent Technologies, Inc., Santa Clara, CA, USA) at a density of 200,000 cells per well in 180 µL of Seahorse XF RPMI assay medium, supplemented with 1 mM pyruvate, 2 mM glutamine, and 5 mM glucose. Mitochondrial respiration was assessed by measuring the oxygen consumption rate (OCR) using the Seahorse T Cell Metabolic Profiling Kit (Agilent Technologies, Inc., Santa Clara, CA, USA), with sequential injections of 1.5 µM oligomycin, 2.5 µM BAM15, and 0.5 µM rotenone/antimycin A. Glycolytic activity, reflected by the extracellular acidification rate (ECAR), was evaluated using the Glycolytic Rate Assay (Agilent Technologies, Inc., Santa Clara, CA, USA), with the sequential injection of 0.5 µM rotenone/antimycin A and 50 mM 2-deoxy-glucose. All conditions were analyzed in triplicate, and results are presented as means of individual experiments.

### 2.9. Intracellular Quantification of β-Hydroxybutyrate

The quantification of intracellular ketone body concentrations was performed using the β-Hydroxybutyrate (Ketone Body) Colorimetric Assay Kit (Cat. No. 700190, Cayman Chemical, Ann Arbor, MI, USA) as instructed by the manufacturer. In brief, PBMC were isolated at defined time points after the intake of exogenous ketone bodies or after in vitro incubation with 10 mM beta-hydroxybutyrate (BHB, (±)-3-Hydroxybutyric acid sodium salt; Sigma-Aldrich, St. Louis, MO, USA). The isolated PBMC were resuspended in assay buffer at a concentration of 12 × 10^6^ cells/mL, followed by physical lysis via sonication. After centrifugation, the supernatant was collected. Absorbance was measured at 450 nm using the FilterMax F3 microplate reader.

### 2.10. Quantification of Metabolic Parameters

Serum concentrations of glucose were quantified by kinetic colorimetric assays (GLUC3 kit; Roche Diagnostics, Indianapolis, IN, USA, 05168791190) on a Cobas 8000/c702 system (Roche Diagnostics). Quantification of serum insulin and c-peptide have been performed by an electrochemiluminescence immunoassay (ECLIA) using the Insulin kit (Roche Diagnostics, #07027559190) and the C-Peptide kit (Roche Diagnostics, #7027168190) on a Cobas 8000/e801 system (Roche Diagnostics) according to the manufacturer’s instructions.

### 2.11. Assessment of Quality of Life and Fatigue Severity

The World Health Organization’s Quality of Life Assessment (WHOQOL-BREF), the Short Form Health Survey (SF-36), and the Fatigue Assessment Scale (FAS) were applied as previously described [12].

### 2.12. Statistical Analyses

Unless otherwise specified, statistical analyses were conducted using GraphPad Prism version 10 (GraphPad Software, LLC, Boston, MA, USA). Data sets were tested for normal distribution using the D’Agostino & Pearson and Shapiro–Wilk test. Depending on data distribution, either a paired *t*-test or a Wilcoxon matched-pairs signed rank test was applied for comparisons. Results are presented as mean ± standard error of the mean (SEM) or as box plots displaying the median, interquartile range (25th and 75th percentiles), and full data range, unless stated otherwise. To account for multiple testing, the Benjamini–Hochberg correction was applied. Statistical significance was defined as *p* < 0.05: * *p* < 0.05, ** *p* < 0.01, *** *p* < 0.001, and **** *p* < 0.0001.

## 3. Results

### 3.1. Ketone Supplementation Induces Transiently Enhanced Plasma Ketone Levels Without Affecting Body Composition or Quality of Life

**Figure 1:**
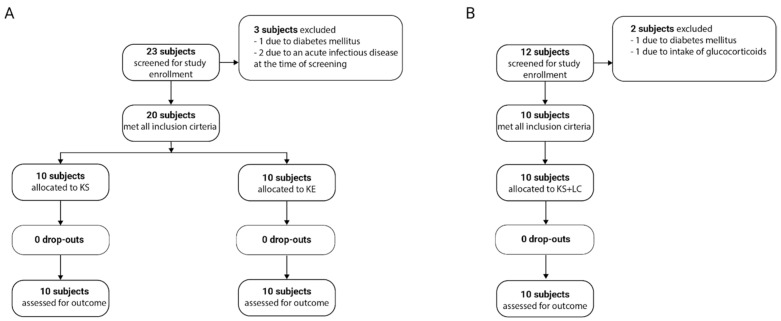
CONSORT recruitment flow diagram. (**A**) CONSORT diagram for intake of KS or KE over three weeks without changes to the dietary habits. (**B**) CONSORT diagram for participants with three weeks of KS intake combined with a low-carbohydrate diet. KS = ketone salts, KE = ketone ester, LC = low-carbohydrate diet.

The effects of supplementation with exogenous ketone bodies, administered as either ketone salts (KS) or ketone esters (KE), were assessed with regard to immunometabolic parameters and quality of life. For this purpose, a total of 23 volunteers were screened for study eligibility, of whom 20 were enrolled. The demographic characteristics of the participants are summarized in Table 1. Participants were assigned to two groups, with 10 individuals receiving KS and 10 receiving KE. No participants dropped out during the intervention period (Figure 1A). Adverse events were reported in both intervention groups. Nausea, diarrhea, and heartburn occurred following both KS and KE intake, but were more pronounced after KS intake. The main adverse event following KE was dizziness, although gastrointestinal side effects were also observed (Supplemental Figure S1).

**Figure 2:**
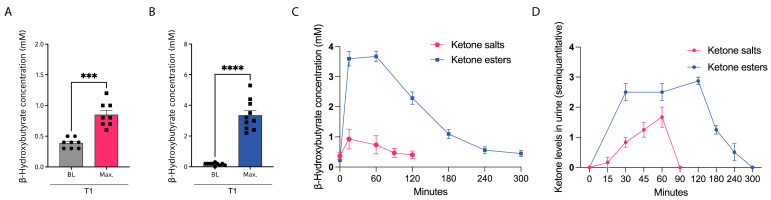
Healthy subjects underwent a three-week supplementation with either ketone salts (KS, red) or ketone esters (KE, blue). (**A**,**B**) Baseline (BL) and maximum (max.) β-hydroxybutyrate (BHB) levels following intake of a single serving of KS (**A**) or KE (**B**), measured using point-of-care testing, after three weeks of supplementation (T1). (**C**) Mean ± SD of blood β-hydroxybutyrate (BHB) concentrations over time across all participants following a single serving of KS or KE. (**D**) Semiquantitative assessment of urinary ketone bodies over time following a single serving of KS or KE with color-coded test strips in healthy subjects after intake of KS or KE. Kinetics were assessed at the beginning of the supplementation period. *** *p* < 0.001, **** *p* < 0.0001 with FDR < 0.05. *n* = 10/10.

Intake of KS induced a rapid rise in plasma ketone body concentrations, increasing from a baseline level of 0.34 ± 0.03 mmol/L to a peak of 1.42 ± 0.11 mmol/L, typically observed 15 min post-ingestion. Thereafter, blood β-hydroxybutyrate (BHB) concentrations declined swiftly, returning to baseline within 2 h (Figure 2A,C). In contrast, ingestion of KE produced a markedly greater elevation in plasma ketone concentrations, rising from a baseline of 0.19 ± 0.03 mmol/L to a mean peak of 5.30 ± 0.34 mmol/L between 15 and 60 min after intake. Notably, in the KE group, BHB levels remained elevated for at least 1 h and returned to baseline only after 4 to 5 h (Figure 2B,C). Semiquantitative analysis of urinary ketone bodies further demonstrated a prolonged duration of ketonuria following KE supplementation compared with KS (Figure 2D).

The question of whether exogenous ketone body supplementation should be regarded as an additional source of caloric intake remains unresolved. In the present study, participants maintained their habitual diets without dietary restrictions. To exclude potential adverse effects on body composition, detailed bioimpedance analyses were conducted. No significant alterations were observed in fat mass, fat-free mass, or total body water. Accordingly, both body weight and body mass index (BMI) remained stable over the three-week intervention period with either KS or KE supplementation. We detected a significant increase in T1 phase angle in both groups (Supplemental Tables S3 and S4). Given that only the ketone salt contained substantial amounts of electrolytes (Ca 230 mg, Mg 235 mg, Na 910 mg; each per serving), whereas the ketone ester contained none, this finding suggests that the observed effect was independent of electrolyte intake. Potential effects of KS and KE intake on general well-being were further evaluated using the Short Form Health Survey (SF-36) and the World Health Organization Quality of Life Assessment (WHOQOL-BREF). Neither intervention produced significant changes in overall quality of life or any of its subdomains. While KS intake had no measurable effect on Fatigue Assessment Scale (FAS) scores, KE supplementation resulted in a significant reduction in fatigue symptoms after three weeks (Supplemental Tables S5 and S6).

### 3.2. Exogenous Ketone Supplementation Does Not Enhance T-Cell Immune Function or Immunometabolism

**Figure 3:**
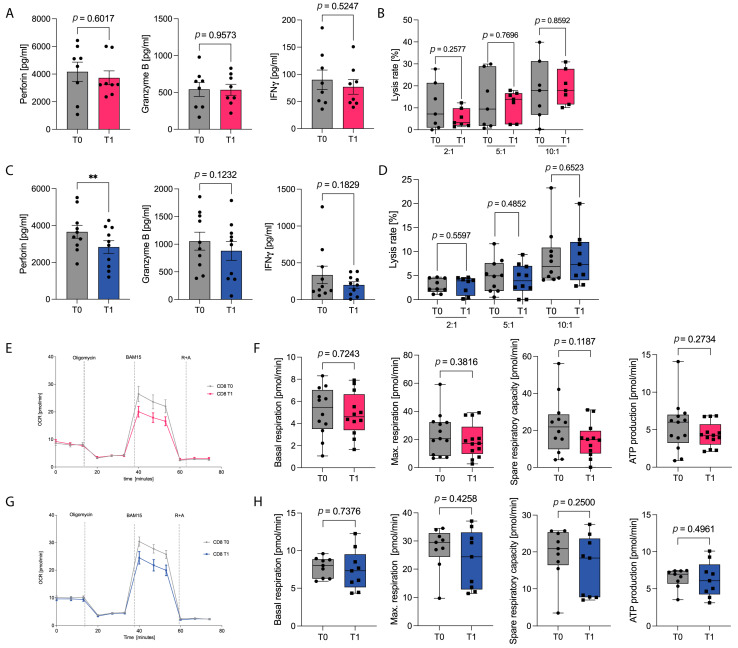
Healthy subjects underwent a three-week supplementation with either ketone salts (KS, red) or ketone esters (KE, blue). Peripheral blood mononuclear cells (PBMCs) were isolated before (T0) and after (T1) the intervention and stimulated ex vivo for 24 h using CD3/CD28 Dynabeads. (**A**,**C**) Cytokine concentrations of perforin, granzyme B, and IFNγ in PBMC culture supernatants, quantified by ELISA, following KS (**A**) or KE (**C**) supplementation. (**B**,**D**) Following isolation, CD8^+^ T cell–mediated cytotoxicity was assessed by a calcein-based fluorescence assay following KS (**B**) or KE (**D**) supplementation. (**E**,**G)** Oxygen consumption rate (OCR) of CD8^+^ T cells following KS (**E**) or KE (**G**) supplementation. (**F**,**H**) Basal respiration, maximum respiration, spare respiratory capacity, and ATP production of CD8^+^ T cells following KS (**F**) or KE (**H**) supplementation, assessed using the Seahorse XFe HS Mini Analyzer. R + A = Rotenon + Antimycin A. ** *p* < 0.01 with FDR < 0.05. *n* = 10/10.

To evaluate the potential effect of KE/KS on T-cell immune capacity, we first assessed activation markers on CD8^+^ T cells. Apart from a small but statistically significant increase in CD38 expression following KS intake, no notable changes were observed in the expression of CD25, CD69, or CD44 on CD8^+^ T cells after either intervention (Supplemental Figure S2). Previous findings have demonstrated that adherence to a ketogenic diet (KD) significantly enhances the cytotoxic CD8^+^ T-cell response in humans. To determine whether exogenous ketone body supplementation exerts similar effects, we next examined the expression and secretion of key CD8^+^ T-cell cytokines. KS intake did not improve mRNA expression or secretion levels of the effector molecules perforin, granzyme B, or interferon-γ (IFNγ). KE intake even resulted in a reduction in perforin secretion, whereas granzyme B and IFNγ expression and secretion remained unaffected (Figure 3A,C and Supplemental Figure S3). Consequently, the overall cytotoxic capacity of CD8^+^ T cells was unchanged following either KS or KE supplementation (Figure 3B,D). Collectively, these data provide no evidence for an enhancement of CD8 T-cell immunity in response to exogenous KS or KE supplementation.

Previous findings have shown that enhanced mitochondrial respiration is the principal mechanism underlying the effects of KD on cytotoxic T cells. To elucidate why exogenously administered β-hydroxybutyrate (BHB) failed to influence CD8^+^ T-cell function, we next performed metabolic profiling of CD8^+^ T cells using Seahorse analyses. Following KS or KE supplementation, activated CD8^+^ T cells exhibited no significant alterations in basal respiration, maximal or spare respiratory capacity, or mitochondrial ATP production (Figure 3E–H). Likewise, glycolytic activity—as indicated by extracellular acidification rate and glycolytic proton efflux rate—remained unchanged (Supplemental Figure S4). Collectively, these findings indicate that transient ketosis induced by exogenous ketone supplementation does not modulate mitochondrial energy metabolism in CD8^+^ T cells.

### 3.3. Combining Exogenous Ketones with a Low-Carbohydrate Diet Does Not Improve Immunometabolism

A KD induces complex metabolic adaptations that extend beyond hepatic ketone body synthesis [11]. In particular, the recurrent plasma glucose peaks and subsequent insulin secretion characteristic of a standard Western diet are largely absent under KD conditions. We therefore hypothesized that ongoing carbohydrate consumption might counteract the potential beneficial effects of exogenous ketone body supplementation. To test this hypothesis, we recruited an independent third cohort of healthy adults who underwent a three-week intervention combining exogenous KS supplementation with a low-carbohydrate, non-ketogenic diet, providing an advised carbohydrate intake of 20–30% of total daily energy (Figure 1B). Actual carbohydrate intake, as assessed by dietary recalls, is shown in Supplemental Table S1. As intended, the low-carbohydrate diet did not elicit endogenous ketone body production, and post-intake BHB concentrations following KS supplementation were comparable to those observed in the group without dietary modification (Supplemental Figure S5). Adverse effects associated with ketone supplementation were less pronounced under low-carbohydrate conditions compared with the standard diet group (Supplemental Figure S1). Serum glucose showed a noticeable but non-significant reduction. Insulin and C-peptide levels remained unchanged, as in the KS and KE groups (Supplemental Figure S6). Bioimpedance analyses revealed a modest but statistically significant reduction in body weight, fat fraction, and BMI, accompanied by an increase in phase angle (Supplemental Table S7). No changes were observed in quality-of-life parameters (Supplemental Table S8). Collectively, ketone supplementation, even when combined with reduced carbohydrate intake, neither enhanced CD8^+^ T-cell immune capacity (Supplemental Figure S7) nor altered mitochondrial metabolism (Supplemental Figure S8).

### 3.4. Exogenous Ketone Supplementation Leads Only to Limited and Short-Lived Intracellular BHB Uptake

**Figure 4:**
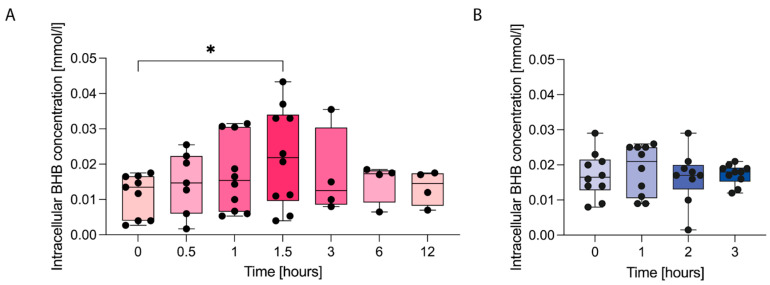
Intracellular β-hydroxybutyrate (BHB) quantification in peripheral blood mononuclear cells (PBMC) after a single intake of (**A**) ketone salts or (**B**) ketone esters. PBMC were isolated at defined time points following oral intake, and intracellular BHB concentrations were determined using the colorimetric BHB assay kit. * *p* < 0.05 with FDR < 0.05. *n* = 4–9/10.

To elucidate the underlying reasons for these unexpected findings, we next examined whether immune cells are, in general, capable of taking up ketone bodies following KS or KE supplementation. In vitro incubation of PBMC with laboratory-grade β-hydroxybutyrate sodium salt, as used in several previous cell culture studies, resulted in a significant intracellular accumulation of BHB, with elevated intracellular BHB levels persisting for up to 12 h (Supplemental Figure S9). Correspondingly, analysis of intracellular BHB concentrations in PBMC from study participants after a single oral dose of KS or KE revealed that ketone salt supplementation induced a stepwise and significant increase in intracellular β-hydroxybutyrate levels, which, however, returned to baseline within 6 h. Surprisingly, KE ingestion did not alter intracellular BHB concentrations in PBMC (Figure 4 A,B).

## 4. Discussion

The ketogenic diet (KD) has gained increasing attention as a powerful regulator of human immunity. Both KD and ketone bodies have been shown to attenuate innate metaflammation while strengthening CD8^+^ T-cell immunity through metabolic reprogramming toward enhanced mitochondrial oxidative phosphorylation [8,9,12,17]. However, the strict dietary constraints associated with KD compromise long-term adherence and pose logistical challenges for clinical implementation.

Exogenous ketone supplementation has therefore been proposed as an alternative means of elevating circulating ketone body concentrations without the need for extensive dietary modification [18,19,20]. Previous studies have reported reductions in glucose and insulin levels following ketone intake, particularly in overweight individuals with impaired glucose tolerance, suggesting potential therapeutic utility in insulin resistance and type 2 diabetes [13,15,21]. Likewise, beneficial cardiovascular effects and effects on metabolic markers have been described [22,23,24]. Regarding the innate immune system, exogenous BHB inhibited NLRP3 and reduced IL-1β in mouse models [25,26]. In humans, effects may depend on baseline inflammation: ketone esters increased NLRP3/IL-1β in healthy subjects but reduced caspase-1 activation in obesity [21,27,28]. Yet, the impact of oral ketone supplementation on human adaptive immunometabolism has not yet been elucidated.

In order to address this question, we initiated a prospective clinical study enrolling healthy volunteers to consume commercially available exogenous ketone bodies either as ketone salts (KS) or ketone esters (KE) over a period of three weeks. In both groups, the intake of exogenous ketones led to a significant increase in plasma BHB levels. A single dose of ketone salts in which BHB is bound to sodium, magnesium and calcium provided 12.6 g of BHB. A single intake of one serving induced peak BHB concentrations comparable to those observed during a ketogenic diet. However, ketone plasma levels quickly declined to baseline. In contrast, one dose of the ketone ester provided 25 g of BHB. This monoester is composed of BHB and 1,3-butanediol. Upon hydrolysis, BHB is released directly, while 1,3-butanediol is hepatically oxidized, thereby generating additional BHB [29,30]. Oral ketone ester administration resulted in mean peak plasma BHB concentrations well above those on average usually attained during a ketogenic diet [11,31,32]. Although persisting longer than plasma levels induced by ketone salts, the effect remained transient, declining below the ketosis threshold within four hours of ingestion.

Interestingly, KE intake was associated with a reduction in fatigue symptoms, echoing earlier reports suggesting beneficial effects of ketone bodies on cognitive and psychological well-being [33,34]. However, as participants were healthy and exhibited no pathological fatigue at baseline, placebo effects cannot be excluded. The distinct taste and texture of KS compared with KE precluded blinding and placebo control. Therefore, subjective outcome measures should be interpreted with caution. The observed increase in phase angle was independent of electrolyte content of KS/KE, but may relate to indirect changes in hydration or intracellular electrolyte balance, which could alter extracellular ion composition and electrical conductivity, thereby reducing bioimpedance resistance [35,36].

Contrary to expectations, ketosis induced by KS or KE supplementation did not modulate CD8^+^ T-cell cytokine secretion, immune function, or mitochondrial metabolism, even when considering a potential impact of ex vivo cell culture on primary human CD8^+^ T cells [37]. This finding contrasts with previous work demonstrating that endogenously induced ketosis via KD enhances CD8^+^ T-cell immunity through metabolic reprogramming toward oxidative phosphorylation [8,9].

To explain this discrepancy, it might be reasonable to assume that not only BHB concentrations may contribute to the immunometabolic effects of KD but also extensive changes in macronutrient composition and systemic metabolism entailed by KD, leading to broad alterations in the serum metabolome, including lipid mediators, amino acid profiles, and redox balance, that may act synergistically to modulate adaptive immunity [11]. Exogenous ketone supplementation raises circulating BHB in isolation, leaving glucose and insulin dynamics largely unchanged. Continued postprandial glucose and insulin fluctuations could perpetuate inflammasome activation and thereby counteract potential immunometabolic benefits [38,39,40]. However, we could refute this hypothesis, as combining KS intake with a low-carbohydrate, non-ketogenic diet, intended to attenuate glycemic excursions, did not restore immunomodulatory effects despite modest reductions in body weight and fat mass.

As these findings were largely unexpected, we examined cellular uptake as a potential limiting factor. Intracellular BHB concentrations in PBMC did not increase after KE consumption, which may explain the absence of immunological effects. In contrast, BHB-sodium was readily taken up by PBMC both after KS intake and following in vitro exposure, consistent with previous ex vivo data [8,9]. To further corroborate these findings, future studies should also determine the expression of ketolysis enzymes (SCOT, BDH) under sustained nutritional ketosis compared to short-term exposure to KS or KE [41]. In addition, BHB uptake in purified CD8^+^ T-cells might be different from PBMC and remains to be investigated.

Together, these results point to formulation-dependent differences in cellular BHB bioavailability, potentially driven by distinct transport kinetics, hydrolysis dynamics, or other pharmacokinetic factors. However, since BHB was detectable in PBMC for only ~3 h post-ingestion, the lack of measurable effects may indicate that sustained exposure, similar to that achieved by a ketogenic diet or prolonged in vitro incubation, is required for ketone-mediated immunomodulation.

Higher or more frequent dosing, particularly for KS, might therefore be necessary to elicit measurable effects. Yet, gastrointestinal side effects were already evident at the applied doses, which adhered to the manufacturer’s recommendations, making further dose escalation difficult to implement in practice.

## 5. Conclusions

In conclusion, our data show that supplementation with exogenous ketone preparations did not replicate the beneficial effects on cytotoxic T lymphocyte immunometabolism reported for endogenously induced ketosis. Nevertheless, developing modified formulations with more favorable pharmacokinetic and pharmacodynamic profiles may be worthwhile to enable immunomodulation by exogenous ketones as an alternative to strict adherence to a ketogenic diet.

## Figures and Tables

**Table 1:** Baseline characteristics of participants in the respective study groups receiving ketone salts (KS), ketone esters (KE), or ketone salts combined with a low-carbohydrate diet (KS-LC).

	KS	KE	KS-LC
Mean age [years]	27.1	28.6	38.9
Age distribution [years]	22–47	22–48	24–58
Sex [male/female/divers]	7/3/0	6/4/0	9/1/0
Body weight [mean; range, kg]	70.5 [53.4–84.9]	66.5 [48.8–79.0]	71.9 [60.7–98.1]
BMI [mean; range, kg/m^2^]	22.0 [20.4–24.0]	21.6 [18.2–24.7]	24.5 [21.5–30.6]

## Data Availability

The datasets used and or analyzed during the current study are available from the corresponding author upon reasonable request.

## Supplementary Materials

The following supporting information can be downloaded at: https://www.mdpi.com/article/10.3390/nu18050778/s1.

## Abbreviations

The following abbreviations are used in this manuscript:

KD	Ketogenic diet
BHB	β-hydroxybutyrate
KS	Ketone salts
KE	Ketone esters
KS-LC	Ketone salts and low-carbohydrate diet
CTL	Cytotoxic T lymphocyte
BMI	Body mass index
SF-36	Short Form Health Survey
WHOQOL-BREF	World Health Organization Quality of Life Assessment
FAS	Fatigue Assessment Scale
PBMC	Peripheral blood mononuclear cells
ELISA	Enzyme-Linked Immunosorbent Assay
OCR	Oxygen consumption rate
ECAR	Extracellular acidification rate
