# Supplementary material for: Effect of Oral Ketone Body Intake on Human CD8+ T-Cell Immunometabolism
Source: Nutrients. 2026 Feb 27;18(5):778. doi: 10.3390/nu18050778 (PMC12987257; doi:10.3390/nu18050778)
Supplement: Supplementary file 1 [file nutrients-18-00778-s001.zip › nutrients-4143105-supplementary.pdf]

Effect of Oral Ketone Body Intake on Human $CD8^+$ T-Cell Immunometabolism

David Effinger [1,2], Simon Hirschberger [1,2], Thore Arntjen [2], Michaela Zell [2], Lesca Miriam Holdt [3] and Simone Kreth [1,2,*]

[1] Research Unit Immune Function and Immune Metabolism, Walter Brendel Centre of Experimental Medicine, Ludwig-Maximilian-University (LMU), 81377 Munich, Germany; david.effinger@med.uni-muenchen.de (D.E.)
[2] Department of Anaesthesiology, LMU University Hospital, 81377 Munich, Germany
[3] Institute of Laboratory Medicine, LMU University Hospital, 81377 Munich, Germany
* Correspondence: simone.kreth@med.uni-muenchen.de

Supplemental material

Supplemental Figures

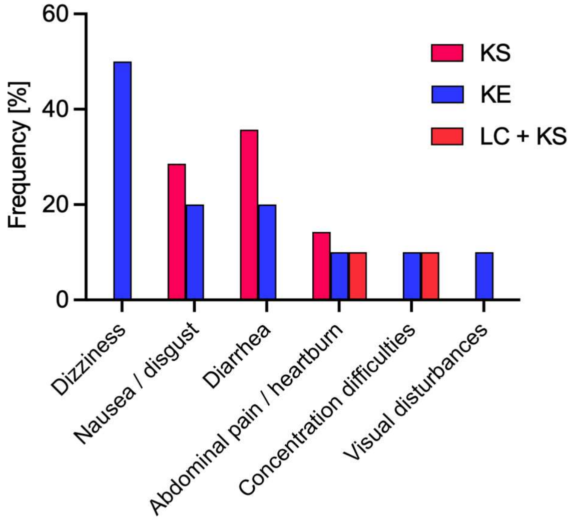

Supplemental Figure S1. Reported adverse events after intake of ketone salts (KS), ketone esters (KE) or ketone salts combined with a low-carbohydrate diet (KS-LC). Bars indicate the percentage of participants experiencing each event. n=10/10.

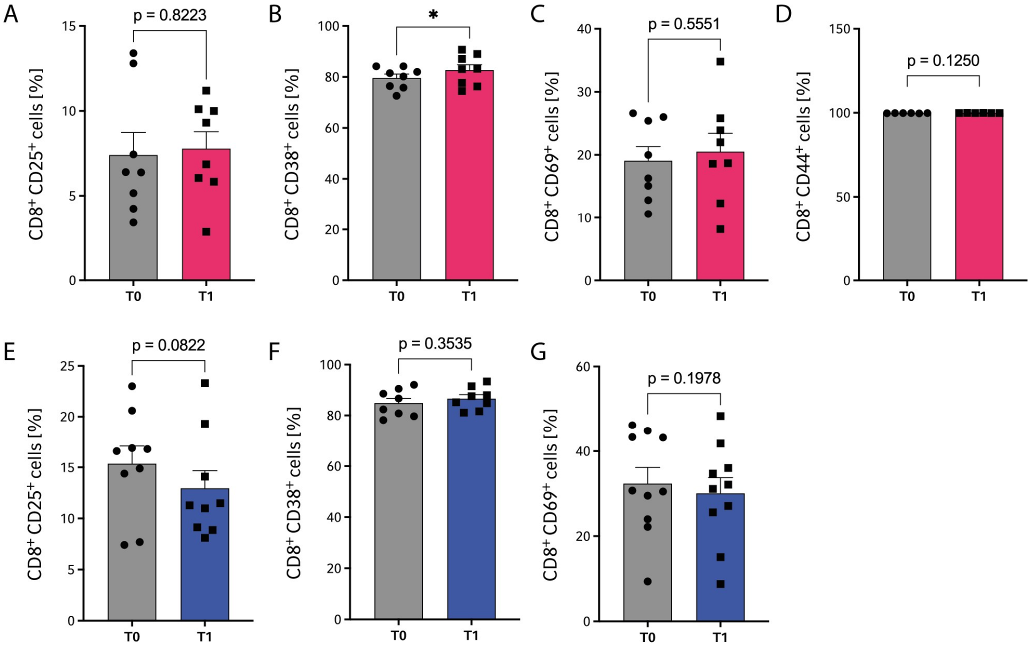

Supplemental Figure S2. Healthy subjects underwent a three-week supplementation with either ketone salts (KS) or ketone esters (KE). Analyses of activation markers CD25, CD38, CD69, and CD44 on CD8+ T cells in participants before (T0) and after (T1) three weeks of supplementation with ketone salts (**A–D**) or ketone esters (**E–G**), assessed via flow cytometry. *p<0.05, *n*=10.

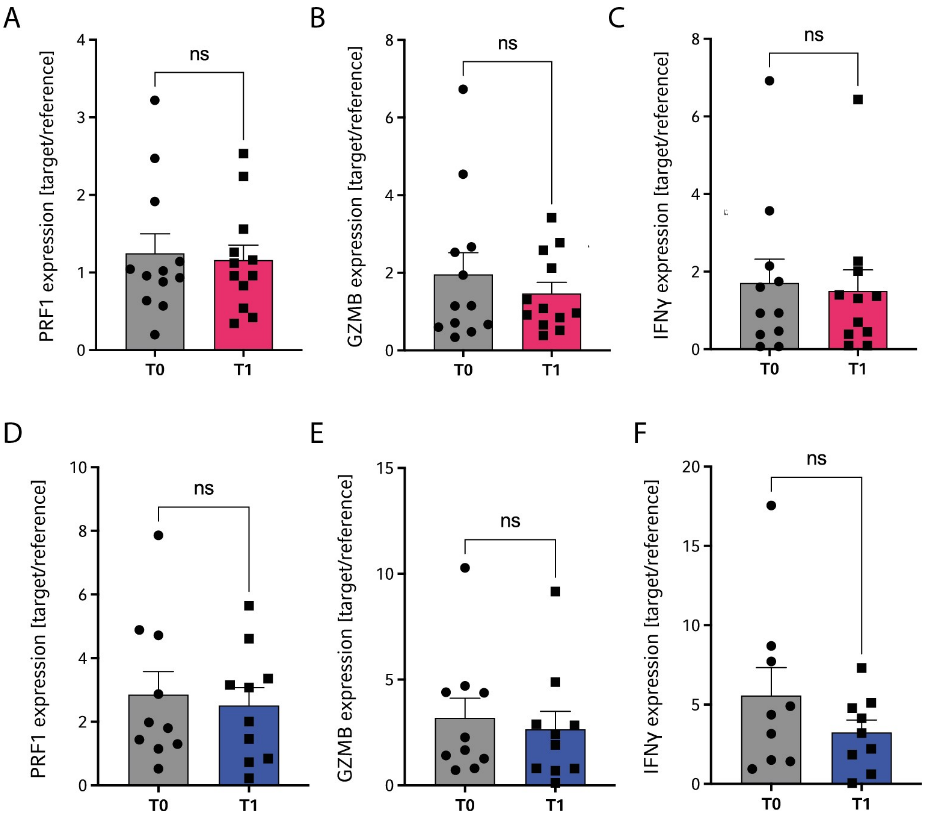

Supplemental Figure S3. Healthy subjects underwent a three-week supplementation with either ketone salts (KS, red) or ketone esters (KE, blue). Peripheral blood mononuclear cells (PBMC) were isolated and stimulated ex vivo for 24 hours using CD3/CD28 Dynabeads. Quantification of mRNA expression of effector cytokines PRF1, GZMB, and IFNγ in $CD8^+$ T cells before (T0) and after (T1) three weeks of supplementation with **A-C**) ketone salts or **D-F**) ketone esters relative to endogenous controls. *n*=10.

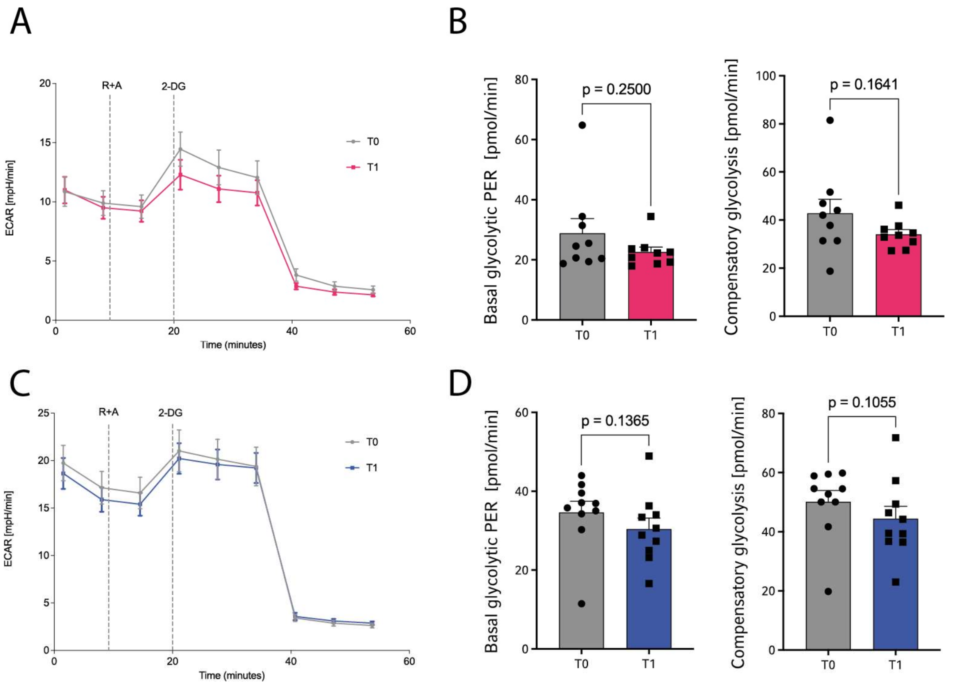

Supplemental Figure S4. Healthy subjects underwent a three-week supplementation with either ketone salts (KS, red) or ketone esters (KE, blue). Peripheral blood mononuclear cells (PBMC) were isolated before (T0) and after (T1) the intervention and stimulated ex vivo for 24 hours using CD3/CD28 Dynabeads. CD8⁺ T cells were isolated using magnetic cell separation. **A, C)** Extracellular acidification rate (ECAR) of CD8⁺ T cells following KS (**A**) or KE (**C**) supplementation. **B, D)** Basal and compensatory glycolytic proton efflux rate (PER) of CD8⁺ T cells following KS (**B**) or KE (**D**) supplementation, assessed using the Seahorse XFe HS Mini Analyzer. n = 9/10.

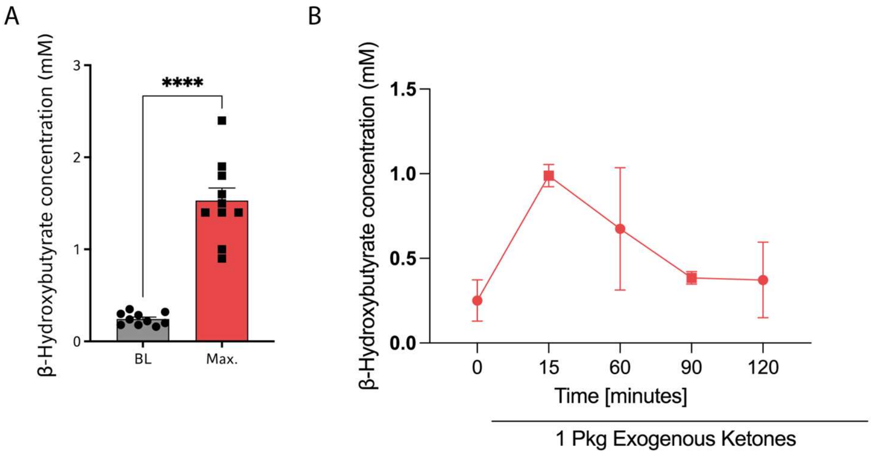

Supplemental Figure S5. Healthy subjects underwent a three-week supplementation with ketone salts combined with a low-carbohydrate diet. **A/B)** Baseline (BL) and maximum (Max.) β-hydroxybutyrate (BHB) levels following intake of a single serving of KS, measured using point-of-care testing. **C)** Blood concentrations of BHB over time following a single serving of KS. ****p<0.0001. n=10.

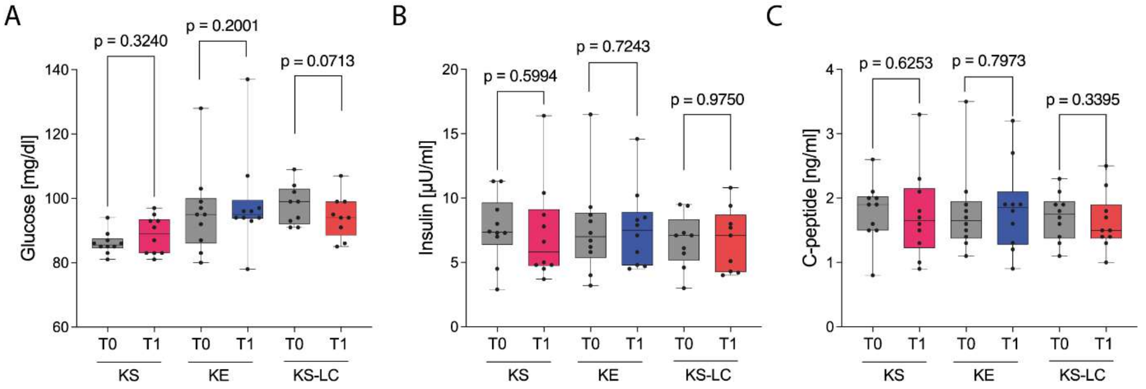

Supplemental Figure S6. Healthy subjects underwent a three-week supplementation with either ketone salts (KS), ketone esters (KE), or a combination of ketone salts with a low-carbohydrate diet (KS-LC). Serum glucose (**A**), insulin (**B**), and C-peptide (**C**) levels were measured before the intervention (T0) and after the three-week supplementation phase (T1). n = 10/10/10.

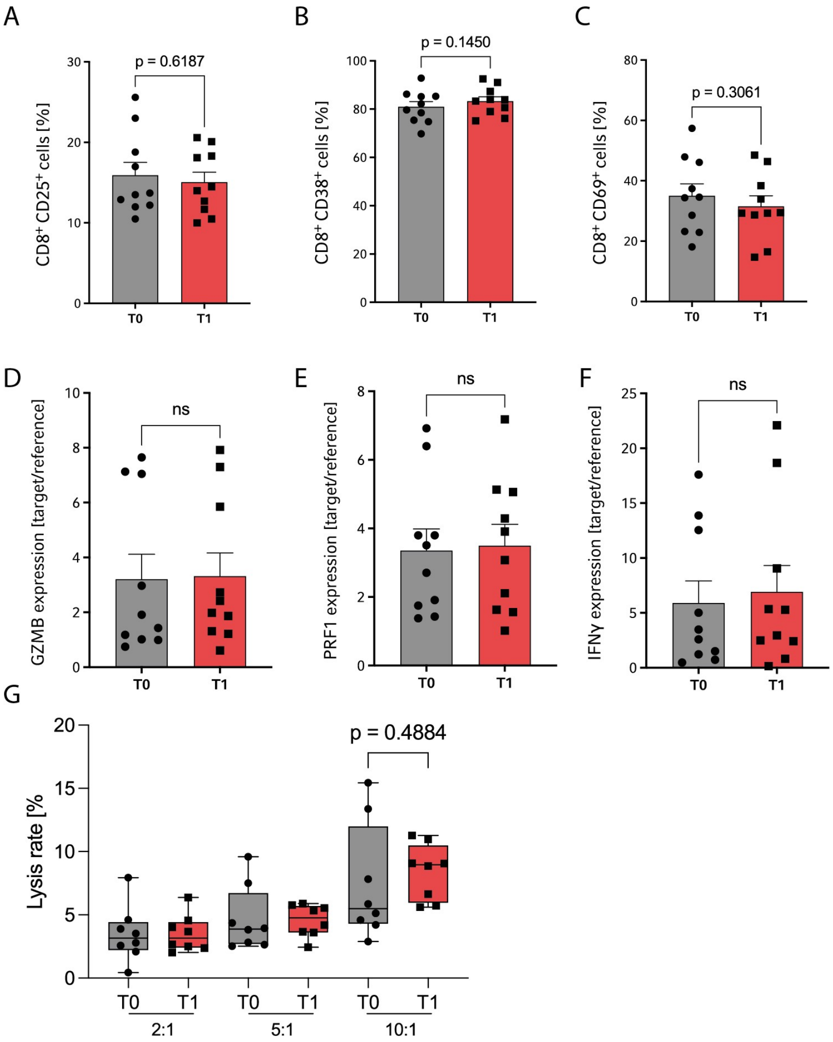

Supplemental Figure S7. Healthy subjects underwent a three-week supplementation with ketone salts combined with a low-carbohydrate diet. Peripheral blood mononuclear cells (PBMC) were isolated and stimulated ex vivo for 24 hours using CD3/CD28 Dynabeads. Respective analyses before (T0) and after (T1) the intervention. **A-C)** Analyses of activation markers CD25, CD38 and CD69 on $CD8^+$ T cells. **D-F)** Quantification of mRNA expression of effector cytokines GZMB, PRF1, and IFNγ in $CD8^+$ T cells. **G)** $CD8^+$ T cell–mediated cytotoxicity assessed by a calcein-based fluorescence assay. n=10.

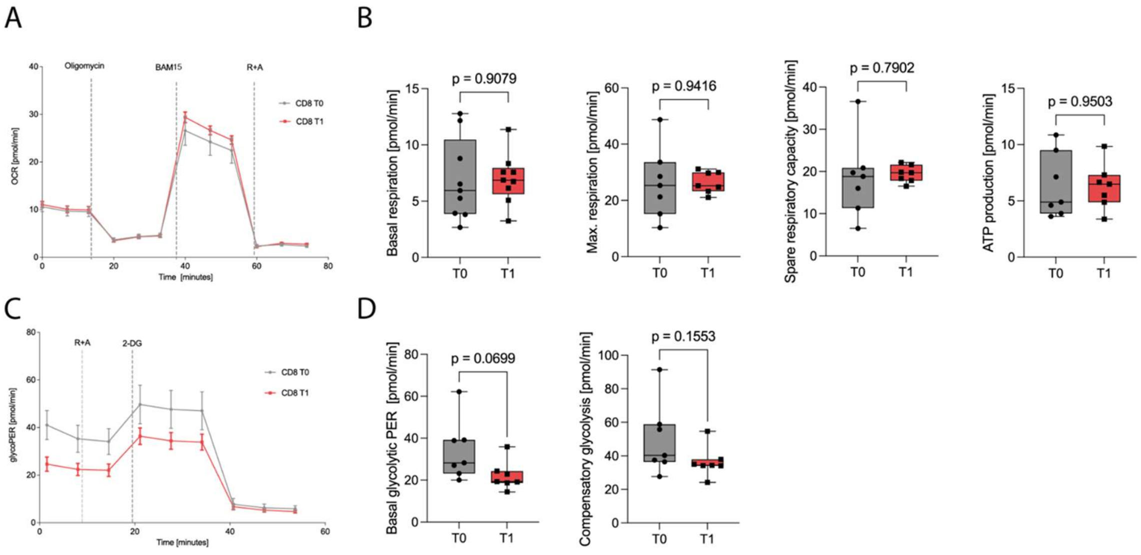

Supplemental Figure S8. Healthy subjects underwent a three-week supplementation with ketone salts combined with a low-carbohydrate diet. Peripheral blood mononuclear cells (PBMC) were isolated before (T0) and after (T1) the intervention and stimulated ex vivo for 24 hours using CD3/CD28 Dynabeads. $CD8^+$ T cells were isolated using magnetic cell separation. Representation of the **A)** oxygen consumption rate (OCR) and **B)** basal respiration, maximal respiration, spare respiratory capacity and ATP production of $CD8^+$ T cells. **C)** Glycolytic proton efflux rate (GlycoPER) and **D)** basal and compensatory glycolytic proton efflux rate (PER) assessed using the Seahorse XFe HS Mini Analyzer. R+A = Rotenon + Antimycin A. n=9.

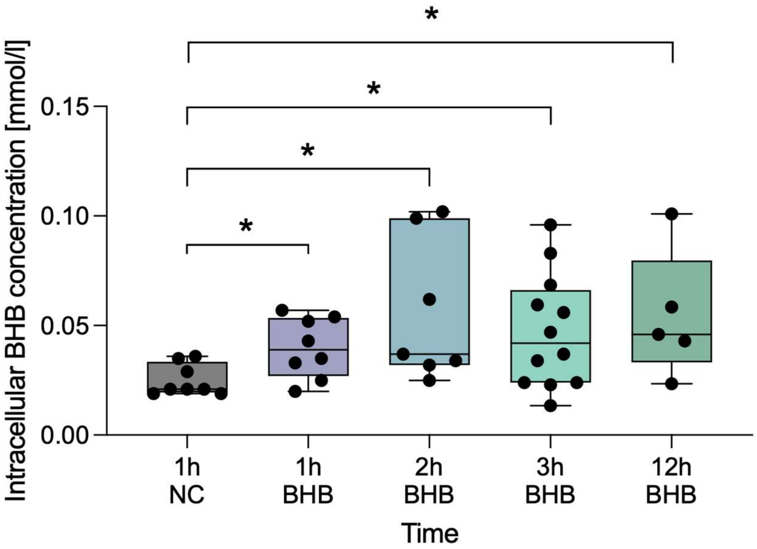

Supplemental Figure S9. PBMC from healthy subjects were incubated in RPMI containing 80 mg/dl glucose in the absence (NC) or presence of β-hydroxybutyrate (BHB) for 1-12 hours. Intracellular β-hydroxybutyrate concentrations in peripheral blood mononuclear cells (PBMC) were determined using the colorimetric BHB assay kit. *$p<0.05$. n=8-12.

Supplemental Tables

Supplemental Table S1. Average daily macronutrient intake during the study, expressed as relative contribution to total energy intake (% of total energy).

	**Participants without dietary changes**	**Participants following low-cabrohydrate diet**
**Carbohydrates [mean ± SEM]**	58.23 % ± 13.46 %	23.23 % ± 2.14 %
**Fat [mean ± SEM]**	28.65 % ± 5.89 %	49.52 % ± 4.22 %
**Protein [mean ± SEM]**	13.12 % ± 2.14 %	27.25 % ± 3.67 %

Supplemental Table S2. qRT-PCR Primer assays

**Target**	**Fluorophor**	**Cat. No.**	
RPL13A	FAM	CEP0052324 12001950qHsa	Bio-Rad Laboratories, Inc. CA, USA
TBP	Cy5	CIP0036255 10031231qHsa	Bio-Rad Laboratories, Inc. CA, USA
PRF1	HEX	CEP0039588 10031228qHsa	Bio-Rad Laboratories, Inc. CA, USA
GZMB	HEX	CIP0029030 10031228qHsa	Bio-Rad Laboratories, Inc. CA, USA
INFɣ	FAM	CEP0050640 12001950qHsa	Bio-Rad Laboratories, Inc. CA, USA

Supplemental Table S3. Body composition and bioimpedance analysis at baseline (T0) and after three weeks (T1) of KS intake

	T0	T1	p-value
**n**	10	10	
**Weight [kg]**	70.48	70.17	0.4874
**BMI [kg/m²]**	22.01	21.90	0.4106
**Fat fraction [%]**	18.91	18.55	0.4436
**Fat mass [kg]**	13.25	13.10	0.6719
**Visceral fat level**	3.58	3.75	0.6250
**Fat free mass [kg]**	57.24	57.12	0.7491
**Bone mass [kg]**	2.86	2.86	>0.9999
**Phase angle [°]**	6.07	6.25	**0.0096**
**Predicted muscle mass [kg]**	54.37	54.24	0.7457
**Total body water [kg]**	40.25	40.26	0.9719
**Extracellular water [kg]**	16.67	16.58	0.3694
**Intracellular water [kg]**	22.68	22.86	0.2212

Supplemental Table S4. Body composition and bioimpedance analysis at baseline (T0) and after three weeks (T1) of KE intake

	**T0**	**T1**	**p-value**
**n**	10	10	
**Weight [kg]**	66.48	66.88	0.1957
**BMI [kg/m²]**	21.64	21.81	0.2029
**Fat fraction [%]**	16.63	16.84	0.6523
**Fat mass [kg]**	11.45	11.74	0.4114
**Visceral fat level**	2.22	2.33	>0.9999
**Fat free mass [kg]**	55.49	55.67	0.5188
**Bone mass [kg]**	2.78	2.80	0.1690
**Phase angle [°]**	5.90	6.01	**0.0212**
**Predicted muscle mass [kg]**	52.71	55.64	0.3185
**Total body water [kg]**	37.73	37.97	0.0907
**Extracellular water [kg]**	16.26	16.19	0.6011
**Intracellular water [kg]**	21.57	21.78	0.0816

Supplemental Table S5. Assessment of health-related quality of life and fatigue symptoms via WHOQOL-BREF and SF-36 questionnaire as well as the Fatigue Assessment Scale in participants following KS intake

		**p-value**
**SF-36 (mean ± SEM), n=10**	**Δ (T1 – T0)**	
- **Physical functioning**	+1.35 % ± 1.35 %	0.3910
- **Physical role functioning**	±0.00 % ± 0.00 %	not calculable
- **Emotional role functioning**	±0.00 % ± 0.00 %	not calculable
- **Bodily pain**	+2.70 % ± 2.70 %	0.3910
- **Mental health**	±0.00 % ± 0.00 %	not calculable
- **Social role functioning**	±0.00 % ± 2.07 %	>0,9999
- **General health perception**	+7.27% ± 5.14 %	0.2522
- **Vitality**	+3.09 % ± 3.09 %	0.3910
**WHOQOL-BREF, (mean ± SEM), n=10**		
- **Physical health**	-0.68 % ± 2.08 %	0.7489
- **Mental health**	-2.70 % ± 2.62 %	0,3242
- **Social relationships**	+0.72 % ± 2.41 %	0.7702
- **Environmental quality**	+1.09 % ± 1.77 %	0.5495
**FAS (mean ± SEM), n=10**		
	+15,00 % ± 8.65 %	0.2254

Supplemental Table S6. Assessment of health-related quality of life and fatigue symptoms via WHOQOL-BREF and SF-36 questionnaire as well as the Fatigue Assessment Scale in participants following KE intake

		**p-value**
**SF-36 (mean ± SEM), n=10**	**Δ (T1 – T0)**	
- **Physical functioning**	±0.00 % ± 0.76 %	>0.9999
- **Physical role functioning**	-2.50 % ± 2.50 %	0.3434
- **Emotional role functioning**	-3.28 % ± 4.76 %	0.5086
- **Bodily pain**	+0.22 % ± 2.42 %	0.9302
- **Mental health**	+3.53 % ± 3.53 %	0.3434
- **Social role functioning**	+0.49 % ± 3.40 %	0.8880
- **General health perception**	+2.61 % ± 3.40 %	0.4620
- **Vitality**	±0.00 % ± 0.76 %	not calculable
**WHOQOL-BREF, (mean ± SEM), n=10**		
- **Physical health**	-0.12 % ± 1.53 %	0.9413
- **Mental health**	-0.65 % ± 3.00 %	0.8332
- **Social relationships**	+3.87 % ± 3.42 %	0.2869
- **Environmental quality**	-1.64 % ± 2.17 %	0.4674
**FAS (mean ± SEM), n=10**		
	-23.00 % ± 8.41 %	**0.0257**

Supplemental Table S7. Body composition and bioimpedance analysis at baseline (T0) and after three weeks (T1) of KS intake combined with a low-carbohydrate diet

	**T0**	**T1**	**p-value**
**n**	10	10	
**Weight [kg]**	71.86	70.51	**0.0195**
**BMI [kg/m²]**	24.53	24.08	**0.0234**
**Fat fraction [%]**	27.93	27.05	**0.0335**
**Fat mass [kg]**	19.88	18.83	0,0547
**Visceral fat level**	4.56	3.67	0,0688
**Fat free mass [kg]**	51.98	51.68	0.1065
**Bone mass [kg]**	2.66	2.64	0.1690
**Phase angle [°]**	5.51	5.66	**0.0391**
**Predicted muscle mass [kg]**	49.36	49.84	0.5424
**Total body water [kg]**	34.46	33.70	0.3179
**Extracellular water [kg]**	15.77	15.43	0.0656
**Intracellular water [kg]**	18.69	18.27	0.8750

Supplemental Table S8. Assessment of health-related quality of life and fatigue symptoms via WHOQOL-BREF and SF-36 questionnaire as well as the Fatigue Assessment Scale in participants following KS intake combined with a low-carbohydrate diet

		**p-value**
**SF-36 (mean ± SEM), n=10**	**Δ (T1 – T0)**	
- **Physical functioning**	±0.00 % ± 0.09 %	>0.9999
- **Physical role functioning**	-5.26 % ± 5,26 %	>0.9999
- **Emotional role functioning**	+9.02 % ± 7.96 %	0.2867
- **Bodily pain**	-1.05 % ± 5.38 %	>0.9999
- **Mental health**	+8.03 % ± 9.97 %	0.7500
- **Social role functioning**	-1.09 % ± 5.18 %	0.8398
- **General health perception**	-0.63 % ± 4.67 %	0.9570
- **Vitality**	+5.74 % ± 8.40 %	0.6250
**WHOQOL-BREF, (mean ± SEM), n=10**		
- **Physical health**	-0.48 % ± 3.23 %	>0.9999
- **Mental health**	-2.12 % ± 5.04 %	0.6839
- **Social relationships**	+1.76 % ± 3.04 %	0.5761
- **Environmental quality**	-1.42 % ± 3.57 %	0.6995
**FAS (mean ± SEM), n=10**		
	+9.18 % ± 13.16 %	0.5046
